# Editorial: Women in science: public mental health 2023

**DOI:** 10.3389/fpubh.2024.1403838

**Published:** 2024-04-10

**Authors:** Yuka Kotozaki

**Affiliations:** Iwate Medical University, Yahaba, Japan

**Keywords:** public health, public mental health, mental health, women's health, women's mental health, global mental health, inequality, gender

This Research Topic is volume III in the series “*Women in Science: Public Mental Health*”. Although women now outnumber men as college graduates, men continue to outnumber women in most science, technology, engineering, and mathematics (STEM) fields and majors (1, 2). There is also a lack of representation of women in senior positions in Public Health (3). According to the UNESCO Institute for Statistics data in 2016, < 30% of researchers in STEM are women (4). In the field of Public Mental Health, there are many highly influential and successful women who are contributing to the field and tackling important questions. This Research Topic focused on female contributions to Public Health, specifically in the field of Mental Health.

After a thorough peer review process, seven manuscripts were accepted for publication. The following is a summary of the main results of each of these manuscripts.

In the first article in this Research Topic, an original research article, Rachubińska et al. examined personality traits and the risk of eating disorders among Polish women. Only the personality trait of neuroticism exhibits a statistically significant effect on the “Cognitive Restraint of Eating,” “Uncontrolled Eating,” and “Emotional Eating” scores. Also, a moderation effect was demonstrated between self-esteem and the personality trait of conscientiousness on the “Cognitive Restraint of Eating” scale score. This suggests that there is a moderating effect between self-esteem and the personality trait of extraversion on the Uncontrolled Eating subscale score.

In the next article, Ding et al. described the correlation between lifestyle behaviors during pregnancy and the postpartum depression (PPD) status of puerpera in the rural areas of South China. A total of 14.6% of women had a PPD status. Women who continued to work during pregnancy had an Edinburgh Postpartum Depression Scale (EPDS) score 1.386 points higher than that of women who did not. For every 1-point increase in the infant feeding-related knowledge score and pregnancy diet diversity score, the EPDS score decreased by 0.188 and 0.484 points, respectively, and for every 1-point increase in the Pittsburgh sleep quality index score, the EPDS score increased by 0.288 points. Age was related to infant feeding-related knowledge (indirect path coefficient = 0.023). During pregnancy, sedentary time was correlated with sleep quality (indirect path coefficient = 0.031) and employment status (indirect path coefficient = 0.043). They suggested that promoting healthy lifestyle behaviors, including reducing sedentary time, improving sleep quality, and increasing dietary diversity, may be effective in reducing PPD occurrence.

In the third article of this topic, a perspective article, Frazier and Doyle advocated for nurturing positive mental health and wellbeing in educational settings using the Preparation and Access, Restoration, Integration, Connection and Community, Educator Support, Strengths-Based Cultivation and Student Voice (PRICES) model. In this article, each component of the PRICES framework is discussed in detail, emphasizing its role in fostering positive health promotion in schools. In addition, examples of implementation that operationalize this model through a collaborative development process focused on the Social, Emotional, and Ethical Learning program are presented.

Jiang et al. examined the effects of virtual exposure to urban greenways (UGW) on mental health. They conducted a randomized controlled experiment to examine the effects of vegetation design along the UGW on stress reduction and attention recovery. Repeated measures ANOVA results indicated that participants experienced increased stress and mental fatigue after the stressor and decreased levels after the UGW intervention. In addition, between-group analyses showed that the shrub and grassland and trees groups had significantly greater stress reduction than the grassland group. They recommended that future UGW designs include diverse vegetation designs, including shrubs or trees, rather than relying solely on grassland.

In the next article, a study protocol article, Hosseini et al. described the impact of the COVID-19 pandemic on the wellbeing, work conditions, and education of early career psychiatrists in the WHO Eastern Mediterranean Region. She and her colleagues conducted a mixed-methods study in three Eastern Mediterranean Region Office (EMRO) member countries: Iran, Egypt, and Tunisia. This included a cross-sectional survey with self-report questions and a qualitative study with individual in-depth interviews. The findings of this study will raise awareness of the working conditions of ECPs within the EMRO region and its member societies, both during the COVID-19 pandemic and beyond.

Guo et al. examined the gender differences in geriatric depressive symptoms in urban China. This study was a nationally representative cross-sectional survey among the Chinese population aged 60 years and over. It was found that 95.69% of the participants had depressive symptoms according to the CESD-10, with no statistically significant gender difference with 52.15% of women and 47.85% of men experiencing these symptoms. Geriatric depressive symptoms were also significantly associated with lack of elder care, living alone, ADL dysfunction, and impaired sensory and communication abilities in both female and male participants. Of note, geriatric depressive symptoms are significantly associated with age, marital status, number of children, and living arrangements only among female participants.

Finally, Grattan et al. used a mixed-methods, longitudinal study to examine whether perceived pressure to breastfeed was associated with depression, suicidal ideation, anxiety, birth trauma, and stress concurrently and 4 weeks later in postpartum mothers. Qualitative breastfeeding experiences were also examined. Results indicated that perceived pressure to breastfeed was associated with increased anxiety, stress, and birth trauma symptoms 4 weeks later. Thematic analysis suggested that this may be due to difficulties in meeting the “breast is best” ideal, beliefs that breastfeeding is part of succeeding as a mother, lack of choice and autonomy in infant feeding decisions, and general lack of support.

To conclude, the editors wish to thank all the authors, reviewers, and editorial board members for contributing to this Research Topic. I hope this Research Topic might inspire future and novel research approaches in the field of mental health.

## Author contributions

YK: Writing – original draft, Writing – review & editing.
